# Personalized adaptive virtual reality experience driven by electroencephalography-based pain recognition

**DOI:** 10.1371/journal.pone.0354510

**Published:** 2026-08-24

**Authors:** Sabrina Al Bukhari, Ahmad Zahran, Anzif Anvaj, Muhammed Hamdan, Ahmed Atif, Jinane Mounsef, Yacine Hadjiat

**Affiliations:** 1 Department of Electrical and Computing Engineering, Rochester Institute of Technology, Dubai, United Arab Emirates; 2 College of Medicine, Mohammed Bin Rashid University of Medicine and Health Sciences, Dubai, United Arab Emirates; University of Marburg: Philipps-Universitat Marburg, GERMANY

## Abstract

**Background:**

Non-pharmacological pain management represents an urgent clinical need. Emerging technologies such as virtual reality (VR) and electroencephalography (EEG)-based artificial intelligence (AI) offer promising avenues for objective pain assessment and adaptive therapeutic intervention.

**Purpose:**

This study aims to develop and validate a real-time, closed-loop EEG-driven VR therapy system that classifies pain levels from brain signals and delivers personalized, avatar-guided therapeutic responses.

**Methods:**

An open-source EEG dataset (51 participants; perception condition; laser-induced pain stimuli rated 0–100) was preprocessed using bandpass filtering, Independent Component Analysis (ICA), and AutoReject. Wavelet-based features (Daubechies-4, 5 levels) were extracted from 1-second epochs and used to train two gradient-boosting classifiers: XGBoost and LightGBM. Predicted pain levels were transmitted via HTTP POST requests to Unreal Engine 5.3.2, where a MetaHuman avatar delivered adaptive therapeutic responses.

**Results:**

LightGBM achieved 97.89% classification accuracy (cross-validation: 95.78% ± 0.82%) and XGBoost achieved 97.25% (cross-validation: 96.09% ± 0.70%) across 11 pain classes (0–10), outperforming all comparable studies in the literature. Real-time avatar responses were demonstrated across three pain categories: Slight (1–3), Moderate (4–6), and Severe (7–10).

**Conclusion:**

The study successfully demonstrates the technical feasibility of a closed-loop EEG-VR pain management system using lightweight machine learning models. The system achieves state-of-the-art pain classification accuracy with fine-grained 11-class granularity.

**Implications:**

This system offers a scalable, drug-free alternative for pain management applicable in clinical and rehabilitation settings. The modular design facilitates future extensions, including emotional state tracking, haptic feedback, and reinforcement learning-based personalization.

## Background

There has been a recent surge in technological advances in pain management, including incorporating tools like virtual reality (VR) and artificial intelligence (AI) with neurophysiological monitoring techniques such as electroencephalography (EEG). These advances provide unique and non-invasive solutions that go beyond pharmaceutical approaches. This review of the literature explores existing research in the following core areas: the use of VR for pain relief, AI-based EEG signal processing and interpretation, combined EEG-VR systems, and limitations in existing research. These findings solidify a framework in developing an adaptive, patient-oriented pain management intervention.

### VR for pain relief

VR has been demonstrated to be a powerful tool in pain management and reduction, as it offers immersive environments that divert the patient’s attention from pain. Hoffman et al. [1] argued that immersive VR can serve as an effective tool for distracting patients from painful stimuli, especially for patients experiencing severe pain, like burn victims. Their study demonstrated a 35–50% reduction in pain, which was supported by fMRI scans and neuroimaging evidence of decreased activity in brain regions associated with pain. Contrary to concerns about VR’s inefficacy at high pain levels, their findings confirm its potency even in such extreme cases. Similarly, Mozgai et al. [2] developed VR therapy for veterans using BraveMind, a VR environment designed for Vietnam veterans. The study showed high receptivity and emotional response, noting the impact of a user-oriented and iterative design. Austin and Siddall [3] reviewed nine studies pertaining to VR and AR for spinal cord injury treatment. Despite limited sample sizes, they concluded that such tools can enhance sensory and motor functions. Goudman et al. [4] conducted a comprehensive review of 41 studies and concluded that VR is effective not only for acute pain but also for improving long-term mobility and functionality of patients. Finally, Pittara et al. [5] evaluated 23 VR studies for cancer-related pain and contended that immersive VR setups improved the patient’s experience and reduced their pain, in comparison to non-immersive VR setups. Collectively, these studies underscore VR’s promising potential as a non-invasive, patient-centered intervention that can address varying pain experiences from acute to chronic. While these papers emphasize the potential of VR for pain relief, recent studies have also investigated how pain can be detected and quantified through coupling EEG and artificial intelligence approaches.

### EEG-based AI for pain classification

EEG signals, when coupled with machine learning and deep learning models, offer promising results in the prediction and classification of pain. A systematic review by Mari et al. [6] underscores the potential of applying machine learning (ML) to EEG data for predicting pain intensity, phenotypes, and treatment responses. Through 44 different studies, it was demonstrated that ML models can achieve a predictive accuracy up to 100% for pain intensity and up to 95.24% for treatment response. Despite the risk of bias due to limited sample sizes and lack of external validation, this review affirms the ability of ML models based on EEG data. However, upon examining recent literature, it is evident that most studies in this field favor deep learning (DL) techniques over conventional ML models. For instance, Chen et al. [7] explored the use of CNNs to predict pain levels in individuals with chronic back pain, achieving an AUC between 0.81 and 0.83. Similarly, Al-Nafjan et al. [8] integrated an EEG-based brain-computer interface (BCI) system with deep learning models. Utilizing wavelet-based time-frequency analysis, the authors trained CNN and RNN models, achieving a 91.84% accuracy for binary classification and an 87.94% accuracy for categorizing pain into low, moderate, and high. Yet, conventional ML models remain relevant when applied rigorously. Mari et al. [9] demonstrated this by applying Random Forest models to classify pain levels, achieving an accuracy of 73.18% during cross-validation, with 68.32% and 60.42% accuracies in two external validation settings. Lastly, Elsayed et al. [10] identified the Alpha band to be a key factor in EEG analysis and trained an ANN to classify four levels of pain, achieving an accuracy of 94.83%. These findings reinforce the prospect of ML in EEG analysis and suggest that despite the increase in DL-focused research, there is still a critical area in improving ML techniques in this field. All in all, these advances imply that EEG-based AI solutions can provide a holistic framework for pain assessment, which can be elevated when integrated with other modalities like VR to create a more adaptable approach for pain relief.

### Integrated EEG-VR systems

Recent research has explored the potential of integrating VR and EEG for pain prediction based on the modulation of the individual’s brain activity. Fu et al. [11] investigated the impact of VR therapy on brain activity using a 64-channel EEG system. Ten adult participants underwent a 30-minute VR-guided meditation therapy, including a post-meditation rest period. The results demonstrate a substantial change in the alpha, beta, theta, and delta bands associated with mindfulness and pain modulation. Similarly, Tran et al. [12] explored how VR modulates brain activity in spinal cord injury (SCI) patients with neuropathic pain. Using EEG data, it was noted that VR exposure increased delta and gamma power while decreasing alpha and theta activity, changes that corresponded to the reported pain relief. Ziabari et al. [13] analyzed the effects of avatar embodiment in VR on chronic pain modulation, along with the changes in brain activity, which are measured with the EEG. The study hypothesized a correlation between pain reduction and increased embodiment, especially changes in the alpha and theta bands. Though the findings of this study were not yet available at the time of writing, the study describes a framework for optimizing VR-based analgesia via EEG. Li et al. [14] examined emerging EEG-VR systems and highlighted their feasibility in applications like rehabilitation, education, and entertainment. The authors also address key challenges and emphasize the need for improved EEG electrodes, multimodal setups, and closed-loop systems. Finally, Bi et al. [15] demonstrated that integrating VR with synchronous transcutaneous electrical nerve stimulation (TENS) can lead to measurable outcomes in EEG activity. The results show that event-related potentials (ERPs) associated with pain processing were significantly lessened, implying a dampened neural response to pain. While these studies emphasize the prospect of EEG-VR systems, there still remain gaps to address, including real-time feedback, personalization, and machine learning integration. All of these areas will be addressed in this study by employing an adaptive approach to pain therapy.

### Gaps in existing research

While existing research signifies the potential of EEG-VR systems for pain therapy, several limitations remain. Firstly, most of the studies are centralized around passive VR exposure, where there is a lack of interaction between the individual and the virtual environment. This overshadows the potential of real-time, conversational agents, such as avatars that act like virtual therapists, that can provide adaptive responses based on the physiological state of the patient. A virtual agent can communicate and guide the patient through some meditative practices to minimize their pain. Secondly, while deep learning models have proven to be effective in pain classification and prediction tasks, they are computationally expensive. Deep learning models typically rely on huge volumes of data for training, which makes them less practical for clinical implementations, especially in contexts where resources are limited. Many studies overlook the drawbacks and impediments in real-world applications. In contrast, this study addresses these gaps by proposing a machine learning framework that utilizes EEG data for pain classification and integrates with an interactive VR environment. This allows for a personalized and adaptive approach, where a virtual avatar is present and responds accordingly. By focusing on developing a personalized and responsive therapeutic solution, this approach bridges the gap between theoretical research and clinical applications.

### Purpose and objectives

The overarching aim of this study is to develop a real-time, closed-loop, EEG-driven virtual reality therapy system that provides personalized, non-pharmacological pain management through adaptive avatar-guided interventions. The specific objectives of this study are as follows: (1) To train and validate XGBoost and LightGBM models for fine-grained, multi-class pain classification (11 levels: 0–10) from EEG wavelet features; (2) To design an immersive VR environment in Unreal Engine 5.3.2 featuring an emotionally responsive MetaHuman avatar capable of delivering cognitive-behavioral therapy-aligned interventions; (3) To implement real-time communication between the machine learning classification pipeline and the VR engine via a local server and the Unreal Web Server (UWS) Plugin; and (4) To demonstrate the feasibility of the complete closed-loop system through a functional prototype that maps EEG-predicted pain levels to distinct avatar behavioral states.

### Manuscript organization

The rest of the paper is structured as follows. The *Materials and Methods* section highlights the overall methodology of the study, the system architecture, dataset description, and machine learning pipeline (LightGBM and XGBoost), followed by the design and implementation of the VR environment. Then, the *Results and Discussion* section presents the performance of the machine learning models, in addition to demonstrating the system’s functionality through pain level visualization. The VR blueprint integration and workflow are also described. This section also presents key challenges that were encountered during development and suggests future directions. Finally, the *Conclusion* summarizes the contributions of this study and its implications.

## Materials and methods

This section outlines the materials used and the methods implemented in this study, including EEG preprocessing and data analysis techniques and VR design and configuration.

### Study design

This study employs a computational, cross-sectional experimental design in which a pre-recorded EEG dataset is used to develop and validate machine learning models for pain classification. The system is organized as a closed-loop framework consisting of three sequential stages: (i) EEG data preprocessing, (ii) machine learning-based pain level classification, and (iii) real-time adaptive feedback delivered through an immersive virtual reality environment. This design is consistent with established approaches in EEG-based brain-computer interface research and non-pharmacological pain management system development.

**Fig 1:**
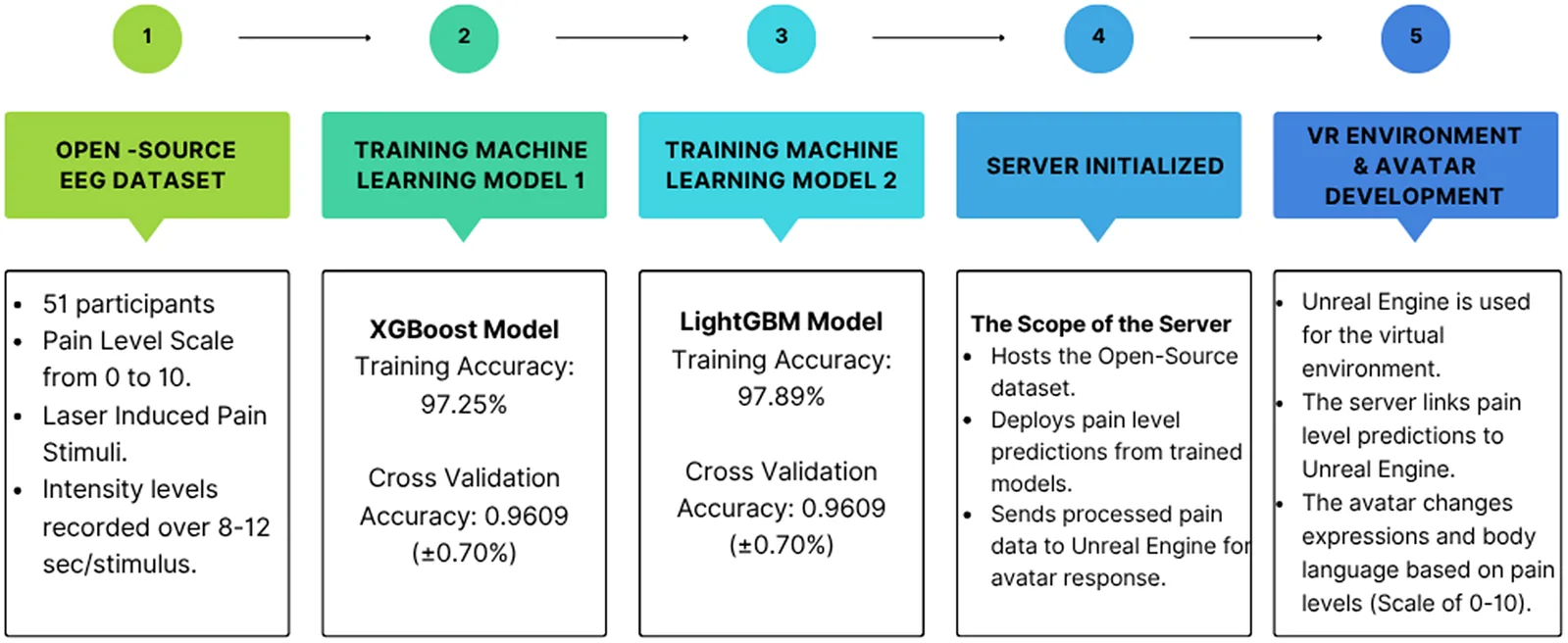
Overall Methodology Flowchart This flowchart highlights the entire pipeline of this study. It begins with EEG data preprocessing, before undergoing machine learning-based pain level classification using XGBoost and LightGBM. Finally, the pipeline culminates in real-time avatar-based reactions within a virtual environment that was developed in Unreal Engine.

Once the models were trained, the models were integrated with the virtual environment through a dedicated server initialized with Unreal Engine. This facilitated the communication between the EEG analysis pipeline and the VR environment and received and transmitted the pain predictions to the simulation in real-time. In the VR program, a human-like avatar was designed to reflect predicted pain levels through changes in facial expressions and body movements, offering an empathetic response to patient discomfort. This methodology is visualized in Fig 1.

### Setting and system architecture

The machine learning pipeline was developed in Python 3.10 using the following libraries: MNE-Python for core biosignal data manipulation [16], PyWavelets for time-frequency multi-resolution decomposition, XGBoost [17] and LightGBM [18] for gradient tree boosting execution, scikit-learn for matrix formatting [19], and SHAP (SHapley Additive exPlanations) [20] for post-hoc additive feature explanations. The virtual reality environment was designed and hosted in Unreal Engine 5.3.2 using the MetaHuman Creator framework for avatar generation, the ConvAI Plugin and web dashboard for dialogue management, and the Unreal Web Server (UWS) Plugin for real-time HTTP-based communication between Python and Unreal Engine. VR deployment was conducted using a Meta Quest 2 headset. The full hardware and software configuration is summarized in Table 1.

**Table 1:** Summary of the hardware and software components used in the study.

Component	Component Identification	Specification (Version/Tool Used)
VR Headset	Hardware	Meta Quest 2
Unreal Engine	Software	Version 5.3.2
Unreal Server Plug-in	Software	UWS Plug-in (Python to Unreal real-time communication)
Machine Learning Models	Software	XGBoost, LightGBM
ConvAI Plugin & Dashboard	Software	ConvAI Platform
MetaHuman Creator	Software	Epic Games MetaHuman Web Creator

**Fig 2:**
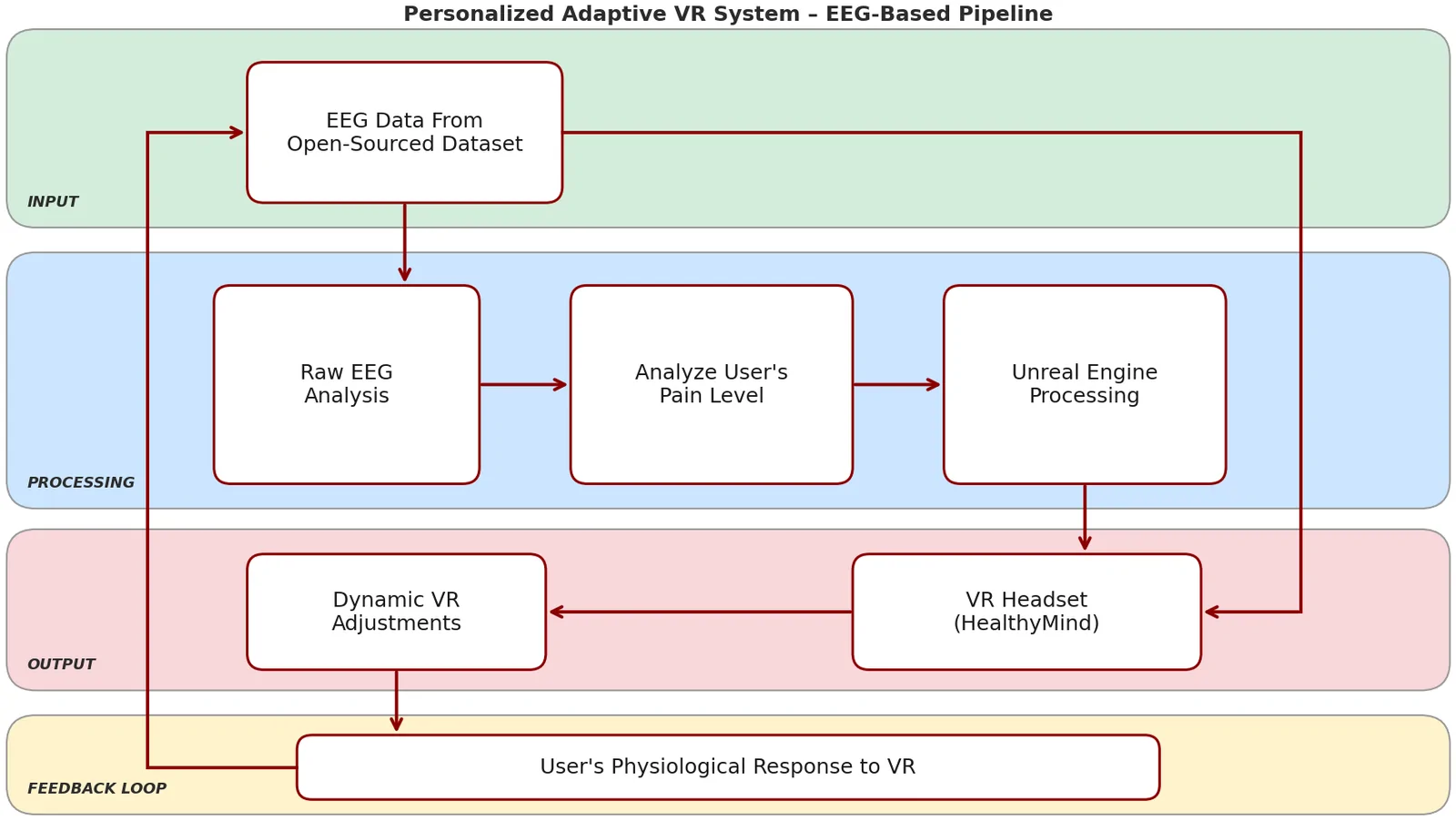
Real-time Feedback Loop for EEG-Based Pain-Responsive VR Therapy Overview of the real-time feedback loop integrating EEG-based pain predictions with VR therapy. EEG signals are analyzed by machine learning models, and predictions are sent to Unreal Engine through a local server. The avatar provides a dynamic response based on the input.

As shown in Fig 2, the system operates as a fully integrated, real-time pipeline in which each component plays a critical role in ensuring seamless therapeutic feedback based on brain signal input. Once the EEG data is obtained, the signal is preprocessed and passed to the machine learning models—XGBoost and LightGBM—which analyze the extracted features to predict the user’s current pain level. These predictions are sent from the Python environment to a local server, which acts as a bridge between the backend and the virtual reality engine. This communication is enabled by the Unreal Web Server (UWS) Plug-in that receives the pain data in JSON format via HTTP POST requests and forwards it to the Unreal Engine 5 environment hosting the therapy session.

Inside Unreal Engine, the system’s logic—designed using Blueprint scripting—interprets the incoming pain level and triggers appropriate avatar behaviors in real time. The avatar, built using MetaHuman Creator, dynamically adjusts its animations, expressions, and voice tone to reflect the user’s pain state. For instance, a low pain level might prompt the avatar to offer calm encouragement, while a higher pain level could trigger more urgent verbal support and intensified facial expressions. The dialogue delivered by the avatar is controlled through the ConvAI Plugin and Web Dashboard, which are pre-configured with pain-sensitive therapeutic responses aligned with cognitive-behavioral therapy (CBT) principles. This full-loop interaction—starting from real-time EEG signal capture, moving through AI-based pain classification, and culminating in responsive VR feedback—ensures that the user experiences a tailored, emotionally intelligent, and immersive therapeutic session. The integration is not only functional but also modular, allowing future extensions such as emotional state tracking or reinforcement learning for personalized therapy evolution.

### Population and dataset

The study utilized the open-access ’Brain Mediators of Pain’ EEG dataset [21], accessed on October 17, 2024. All recordings were fully anonymized prior to public release. The dataset includes recordings from 51 adult participants who underwent laser-induced pain stimulation administered to the left hand at varying intensities. Each recording adheres to the BrainVision Data Exchange Format, comprising three files per session: a header file (.vhdr), a marker file (.vmrk), and a raw EEG data file (.eeg). This structured format expedites preprocessing by explicitly organizing metadata, event markers, and signal data.

The dataset consists of four different experimental conditions—-perception, motor, autonomic, and combined—-each highlighting a different dimension of pain. The conditions were applied in a randomized order to account for sequence effects. In the perception condition, participants were instructed to provide a verbal rating on the pain intensity, using a numerical scale from 0 (no pain) to 100 (worst tolerable pain). This was done three seconds after each stimulus was applied. In the motor condition, reaction time was recorded by having the participants press a button in response to a stimulus. Then, in the autonomic condition, skin conductance responses (SCRs) were recorded as patients focused on the painful stimulus. Lastly, the combined condition integrates all the previous conditions together, meaning participants were asked to respond to the stimuli by pressing a button and rate the pain intensity, and their skin conductance response was recorded. For each condition, 60 painful stimuli were applied to the participants’ left hand at varying pain intensities (low, medium, high). This was done in a randomized order with a time interval ranging from 8 to 12 seconds per stimulus.

All experimental events—-including the stimuli application, button releases, ratings, and SCRs—-were highlighted as event triggers in the .vmrk files. These markers are essential for the preprocessing stage, where data segmentation and label extraction techniques are deployed. For the purpose of this research, only data from the perception condition were utilized. This is because the data is directly relevant to the scope of this research, which is classifying different pain levels. Given the clear structure of the labels and markers, this dataset is ideal for developing models that classify different pain intensities based on EEG data. A summary of the dataset features are provided in Table 2.

**Table 2:** Summary of the EEG dataset used in this study.

Feature	Category	Description
Dataset Format	Format	BrainVision (.vhdr, .vmrk, .eeg)
Number of Participants	Sampling	51 participants per condition
Stimuli per Condition	Stimuli	60 painful stimuli per condition
Pain Intensity Levels	Stimuli	Low, Medium, High
Experimental Conditions	Protocol	Perception, Motor, Autonomic, Combined
Used Condition	Protocol	Perception (verbal pain rating only)
Pain Rating Scale	Labels	0–100 (verbal, 3s post-stimulus)
Stimulus Interval	Timing	8–12 seconds between stimuli
Event Annotations	Metadata	Stimulus, Ratings, Button Presses, SCRs (in .vmrk)

### Sampling strategy and sample size

With 51 participants each receiving 60 stimuli in the perception condition, the dataset yielded 3,060 labeled EEG trials prior to preprocessing. Verbal pain ratings (0–100) were mapped to discrete integer pain levels (0–10) by dividing each raw score by 10 and rounding to the nearest integer, consistent with the original dataset label structure [21]. Following preprocessing and automated epoch rejection via AutoReject, the cleaned feature set was used for model training and evaluation.

A stratified train-test split of 80% training and 20% testing was applied to ensure proportional representation of all 11 pain levels (levels 0–10) across both subsets, preventing class imbalance from biasing model evaluation.

### Model selection

In this study, XGBoost and LightGBM were selected as the primary machine learning models for pain classification. These models were chosen for several reasons. Firstly, EEG datasets tend to be limited in size due to the ethical and practical challenges in data collection. In contrast to deep learning models that require large volume of data for training, XGBoost and LightGBM models can generalize effectively with limited datasets, making them suitable for this task. Secondly, both of these algorithms are computationally efficient. They require less training time, and they are compatible with standard CPU hardware without the need of using GPUs. This is a crucial factor to account for clinical implementations or applications that have limited computational resources. On the other hand, deep neural networks necessitate high computational power, resulting in slower training time. Thirdly, XGBoost and LightGBM have a significant advantage when it comes to interpretability. These algorithms can provide feature importance metrics to better deduce which EEG features are the most influential in pain classification. These insights promote transparency and clinical trust, whereas deep learning models act as “black boxes.” Moreover, XGBoost and LightGBM algorithms are robust enough to handle noisy or incomplete EEG data. This is attributed to their built-in mechanisms for missing values and regularization techniques, which are also used to prevent overfitting. The use of these models in biosignal classification tasks, like EEG analysis, further supports their applicability.

Although it can be observed in recent studies that other classical machine learning models, like Random Forest and Support Vector Machines (SVM), have been used, gradient boosting frameworks generally demonstrate a superior performance. To elaborate, XGBoost and LightGBM models can effectively combine weak learners to enhance their performance, in addition to reducing the possibility of any bias. Compared to Random Forests and SVMs, these algorithms can scale complex features effectively, improve generalization, and offer greater interpretability. Thus, the many advantages offered by XGBoost and LightGBM make them ideal choices for EEG-based pain classification tasks.

Finally, the easy integration of these models with the preprocessing and feature engineering pipelines allows for flexible model development that can be specifically tailored to the needs of clinical assessments. Tables 3 and 4 summarize a comparison of XGBoost and LightGBM with other approaches, like deep learning models, Random Forest, and SVM. This highlights the key factors that motivated the selection of the models presented in this study.

**Table 3:** Comparison of model performance and resource requirements.

Criterion	XGBoost / LightGBM	Deep Learning Models
Data Size Requirement	Small to medium datasets	Large datasets required
Computational Cost	Low to moderate (CPU-friendly)	High (GPU-dependent)
Training Time	Fast	Slow due to model complexity
**Criterion**	**Random Forest**	**SVM**
Data Size Requirement	Small to medium datasets	Small datasets; sensitive to scaling
Computational Cost	Moderate	Moderate to high (kernel-dependent)
Training Time	Slower than boosting models	Slow with large datasets

**Table 4:** Comparison of clinical and practical considerations.

Criterion	XGBoost / LightGBM	Deep Learning Models
Interpretability	High (feature importance)	Low (black-box)
Missing Data Handling	Built-in support	Requires preprocessing
Overfitting Control	Strong regularization options	Requires dropout and tuning
Clinical Deployment	Easy and lightweight	Complex and resource-intensive
Flexibility	Ideal for tabular/engineered data	Best for raw/complex data
**Criterion**	**Random Forest**	**SVM**
Interpretability	High (tree-based importance)	Moderate (kernel-dependent)
Missing Data Handling	Limited; requires imputation	Cannot handle missing data directly
Overfitting Control	Moderate; may overfit with many trees	Sensitive to parameter tuning
Clinical Deployment	Relatively simple	Difficult; tuning-intensive
Flexibility	Good for structured data	Limited by kernel design

### Sample technique and feature extraction

**Fig 3:**
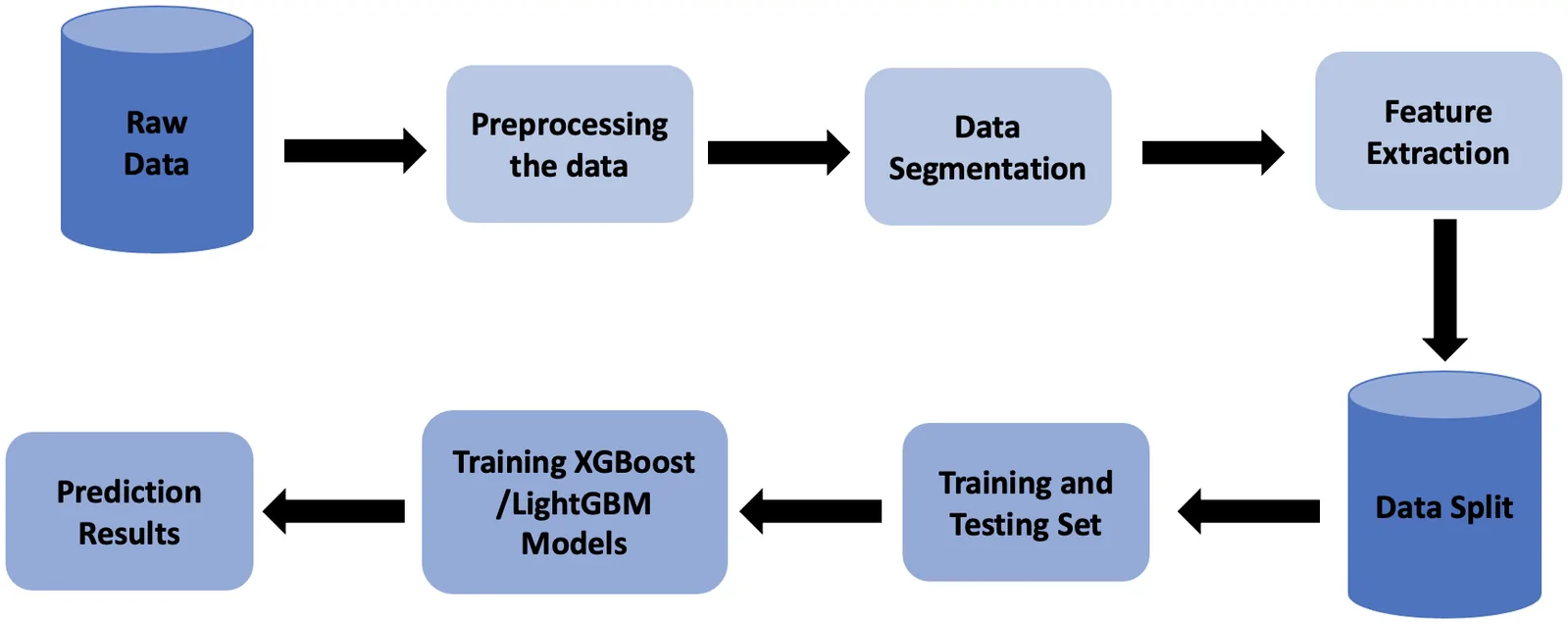
Machine Learning Pipeline for EEG-Based Pain Level Prediction The pipeline includes EEG preprocessing (filtering, ICA, AutoReject), epoch segmentation, wavelet-domain feature extraction, and training of machine learning models for pain level prediction.

The overall machine learning pipeline in this study is summarized in Fig 3. This pipeline highlights the key stages of data preprocessing, feature extraction using wavelet transform, and training/testing two machine learning models for pain level prediction.

**Fig 4:**
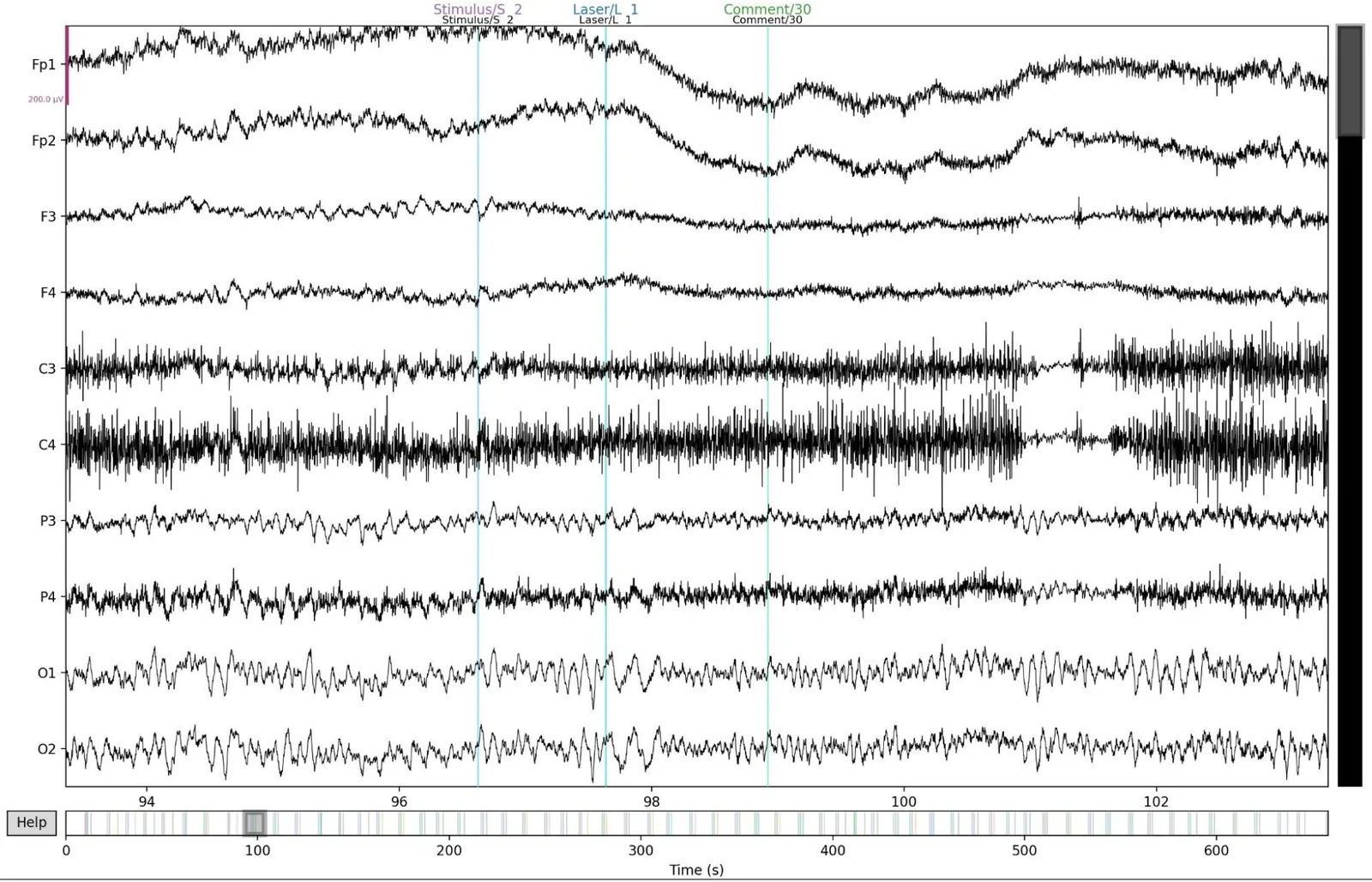
Raw EEG Data Visualization of the unprocessed EEG signal. The plot includes multiple channels showing the original signal before any filtering or artifact removal.

As mentioned earlier, the EEG dataset consisted of recordings from 51 participants, stored in the BrainVision Recorder format. Each recording consists of event markers that correspond to pain levels on a scale from 0 to 10. Using MNE-Python, the raw data with its event markers was visualized, as shown in Fig 4.

**Fig 5:**
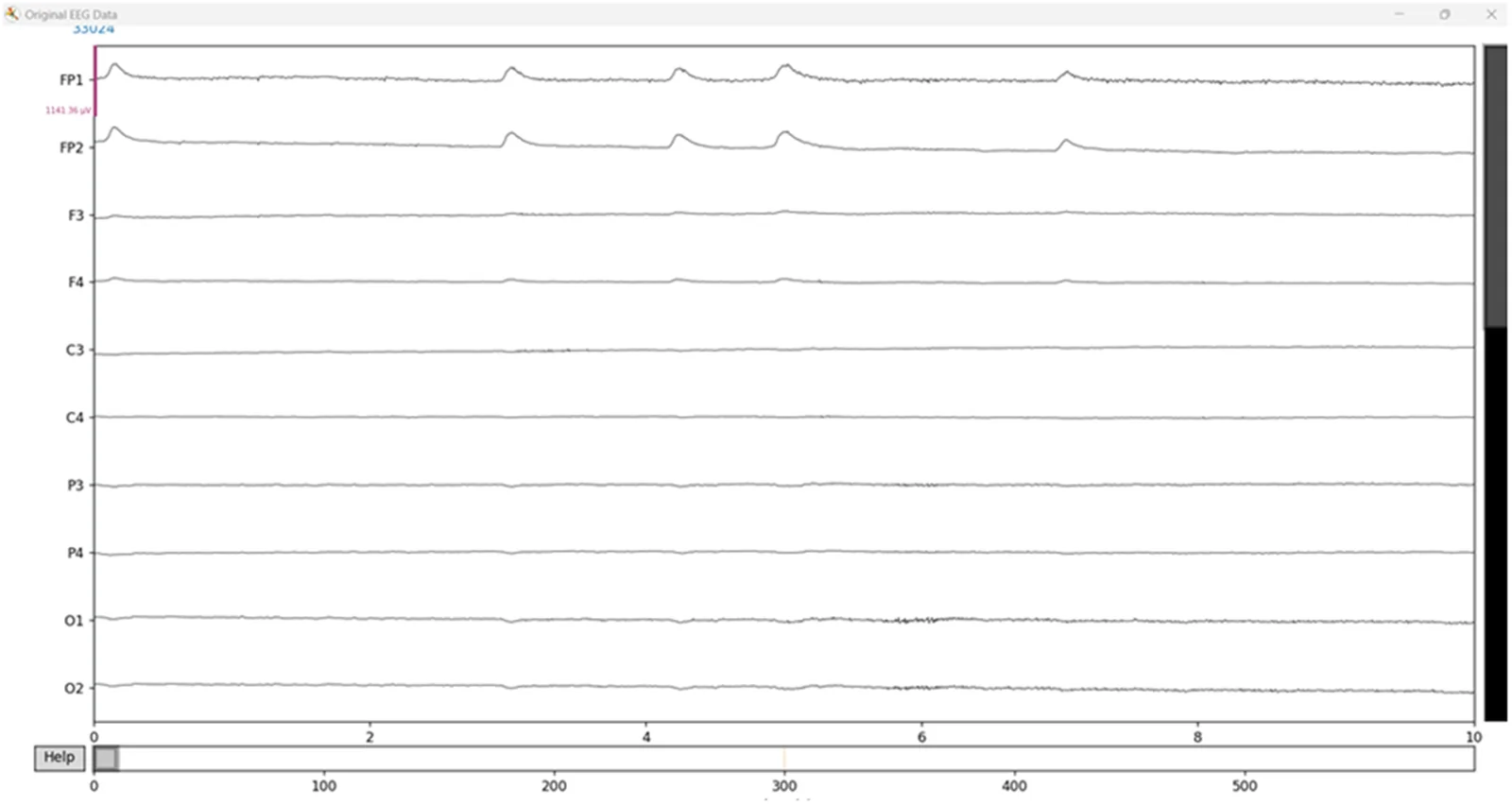
Preprocessed EEG Data EEG signal after preprocessing steps, including bandpass filtering, artifact removal via ICA, baseline correction, and epoching. The cleaned signals provide a more reliable basis for feature extraction and model training.

The EEG preprocessing pipeline is initiated by applying a bandpass filter (1–50 Hz) to eliminate signal noise [22]. Also, the power spectral density (PSD) plot was applied to deduce additional noise that is required to be removed by the notch filter. Then, this was followed by the Independent Component Analysis (ICA), which is used to identify and exclude artifacts such as eye blinks [23]. Baseline correction was applied, and the AutoReject package was used to further clean the data [24]. This package is useful to automatically recognize noisy segments and repair them by interpolating bad channels based on the surrounding data. After cleaning the data, ICA was used again to refit on the good epochs, and the ‘find_bad_eog()’ function was used to compute the occurrence of ocular artifacts so that they would be eliminated from the data as well. The ICA component scores were reviewed to ensure that the relevant brain signals were intact. This combined hybrid cleaning strategy is essential for preventing biosignal data corruption from spectrally overlapping non-cortical artifacts [25]. The final preprocessed data is saved as a ‘.fif’ file. The filtered data is illustrated below in Fig 5.

Using the filtered data, a mapping was created between the event code and labels. Each event or trigger in the data was mapped to a unique integer and stored in the event dictionary. Then, the event markers were visualized using the ‘mne.viz.plot_events()’ function to confirm proper alignment of the labels across time. The data is then segmented into several one-second epochs for feature extraction.

For feature extraction, wavelet transform features were used to capture the characteristics of the signals [8]. Using the ‘pywt.wavedec()’ function, each EEG epoch was decomposed into several levels using the Daubechies-4 (db4) wavelet, where the number of decomposition levels was set to 5. The features extracted with this technique were the mean, variance, energy, maximum wavelet coefficient, minimum wavelet coefficient, median, standard deviation, kurtosis, entropy, and skewness. This provides a robust representation of the EEG signal to identify patterns associated with various pain levels. Finally, the extracted features were compiled and saved as a CSV file with the corresponding pain levels, and this file was used for model training.

### Data analysis

**LightGBM Model**: A multi-class model was created to classify pain levels utilizing the LightGBM algorithm [18]. The initial pain labels were categorized into three classes to reduce complexity and improve model generalizability. Only numerical attributes were preserved and transformed to ‘float32’ format for enhanced memory efficiency. ‘StandardScaler’ was utilized for feature scaling to normalize the distribution and improve the learning process. The dataset was divided into 80% for training and 20% for testing subsets.

Hyperparameter tuning was performed through RandomizedSearchCV utilizing 3-fold cross-validation, refining parameters like ‘num_leaves,’ ‘max_depth,’ ‘learning_rate,’ and ‘n_estimators.’ A backup model was set up with predetermined parameters to guarantee reliability in case of tuning failure. The evaluation of model performance was conducted using metrics such as weighted accuracy, precision, recall, and F1-score. Confusion matrices were created to assess classification results in both absolute numbers and percentage forms.

Additional assessment involved employing ‘classification_report’ for an in-depth analysis of performance across various classes. Feature importance charts created using LightGBM’s integrated tools offered understanding of key features. Moreover, SHAP values were employed to clarify the impact of each feature on the model’s output, guaranteeing interpretability and transparency in the classification of pain levels using EEG data [20].

**XGBoost Model**: To classify pain levels based on EEG data, the XGBoost model was utilized. This algorithm is known for its powerful tree-based ensemble method, subsequently producing high accuracy and speed [17]. The XGBoost classifier was deployed using the ‘xgboost’ Python package. This model was trained based on the features that were extracted from multiple EEG channels, encompassing statistical and wavelet-based descriptors like energy, mean, entropy, and standard deviation. The target variable was a categorical label that referenced different pain levels, indicating that this was a multi-class classification task.

Before modeling, feature smoothing was applied to minimize noise and signal instability. This was done by applying a rolling mean with a window size of 5 for each feature [26]. Once the features in the dataset were smoothed out, the dataset was then split into the input features (X) and target labels (Y). Next, the dataset was stratified and split, such that 80% of it was the training subset, whereas 20% of it was the testing subset. A grid search approach was implemented using cross-validation to optimize the model’s hyperparameters, like maximum tree depth, learning rate, number of estimators, and subsample ratio. Based on this, the following parameters were initialized: a learning rate of 0.1, maximum tree depth of 5, and 200 estimators. This was done to guarantee optimized classification accuracy and reduced overfitting.

The XGBoost model utilized the ‘multi:softmax’ objective function, with the number of output classes set to 11, representing all the unique pain levels identified in the dataset. In addition, 5-fold cross-validation was implemented by randomly shuffling all data points to maximize robustness and minimize overfitting. Finally, the model’s performance was evaluated using various metrics, such as accuracy, precision, recall, and F1-score. Using the test subset, the model was used to generate predictions based on the given data. The predicted labels were then directly compared with the actual values to observe specific occurrences of misclassifications.

### Model validity and reliability

The validity and reliability of the XGBoost and LightGBM models were assessed through complementary strategies. Regarding internal validity, a stratified 80/20 train-test split was implemented to ensure proportional representation of all pain classes across both subsets. Hyperparameter optimization was performed on the training partition using GridSearchCV (5-fold cross-validation for XGBoost) and RandomizedSearchCV (3-fold cross-validation for LightGBM). Regularization parameters like learning rate, maximum depth, and subsampling ratios were tightly tuned to mitigate overfitting, and a StandardScaler was integrated into the LightGBM pipeline to normalize feature distributions.

Regarding reliability, both models demonstrated exceptional stability across evaluation folds, with XGBoost achieving a cross-validation accuracy of 96.09% ± 0.70% and LightGBM achieving 95.78% ± 0.82%. This low standard deviation confirms that the models’ predictive performance is robust and highly insensitive to specific data partitioning. Furthermore, SHAP values and built-in feature importance metrics were leveraged to verify that the models’ decisions are grounded in physiologically meaningful EEG features (e.g., wavelet energy and entropy). This neurophysiological alignment establishes the interpretability and clinical trustworthiness of the classification backend.

Finally, a boundary condition regarding external validity should be noted: the current cross-validation strategy utilizes an epoch-wise (sample-wise) shuffling paradigm. While this approach is highly effective for validating the technical feasibility and real-time responsiveness of the closed-loop VR pipeline, it optimizes performance within the collected dataset pool. To advance this framework toward deployment, future clinical validation will transition to a subject-dependent (leave-one-subject-out) validation architecture to more rigorously evaluate model generalizability across completely unseen patient profiles.

### VR environment design and implementation

As part of the AI-Powered VR Pain Intervention System, the virtual environment was developed in Unreal Engine 5 using the MetaHuman framework. This setup enables a life-like avatar to simulate reactions based on varying pain levels derived from pre-categorized EEG datasets. The system’s primary objective is to offer real-time, immersive feedback to users by representing pain or calm states through avatar behavior. Key areas of focus include data-driven avatar animation, environmental responsiveness, and user interaction within VR [27].

The virtual environment was intentionally kept minimal to direct user focus toward the MetaHuman avatar, who serves as a guide and feedback entity throughout the session. Key features include:

1. A Neutral Outdoor VR Environment: Designed with ambient lighting and minimal distractions.
2. MetaHuman Avatar Placement: Positioned centrally with idle animations for realism.
3. Camera Setup: Includes a player start position to observe the avatar’s reactions.
4. Interaction Zones: Specific regions have sounds, such as river, trees, and grass

**Fig 6:**
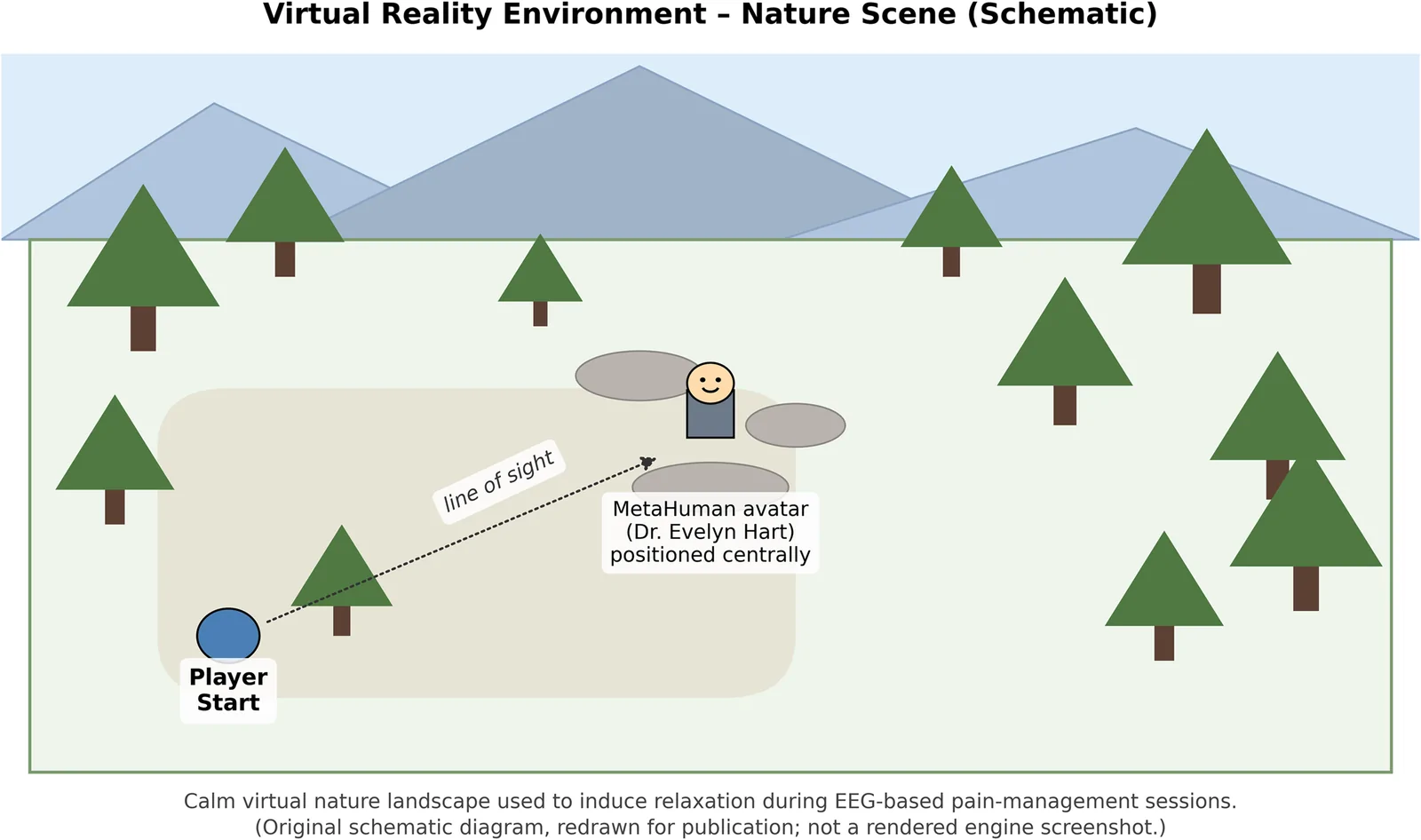
Virtual Reality Environment – Nature Scene A calm virtual nature landscape used to induce relaxation during EEG-based pain management sessions. The user’s perspective includes a virtual avatar, enhancing presence and embodiment within the nature scene. Original schematic diagram; not rendered engine screenshots.

This setup provides a calm and controlled environment ideal for observing emotional and physical cues related to pain simulation. Fig 6 demonstrates how the VR environment looks like, in addition to the virtual avatar immersed in the environment. Because the environment assets (Epic Games’ Electric Dreams sample environment) and the MetaHuman avatar are licensed as Unreal Engine-only content, an original schematic diagram reconstructing the scene composition is shown here in place of direct engine renders.

The avatar, designed using MetaHuman Creator, plays a central role in delivering therapeutic and emotionally responsive interactions. Named Dr. Evelyn Hart, she serves as a virtual therapist who reacts to pain inputs through preprogrammed dialogue and expressions. Her design focuses on realism, empathy, and approachability to foster a calming experience for users.

Key features of the avatar include:

1. Facial Animation and Voice Syncing: The avatar uses expressive facial cues and synchronized voice responses triggered by categorized pain levels.
2. Therapeutic Persona: Dr. Evelyn Hart was given a warm, supportive personality with calm gestures and a soothing tone, designed to comfort users experiencing discomfort.
3. Blueprint-Driven Behavior: Integrated blueprint logic enables the avatar to respond differently based on the incoming pain category—Slight, Moderate, or Severe.
4. Idle and Reactive States: When not responding, the avatar performs subtle idle animations to maintain presence and realism in the environment.
5. An Arabic-speaking avatar for interaction in Arabic language.

**Fig 7:**
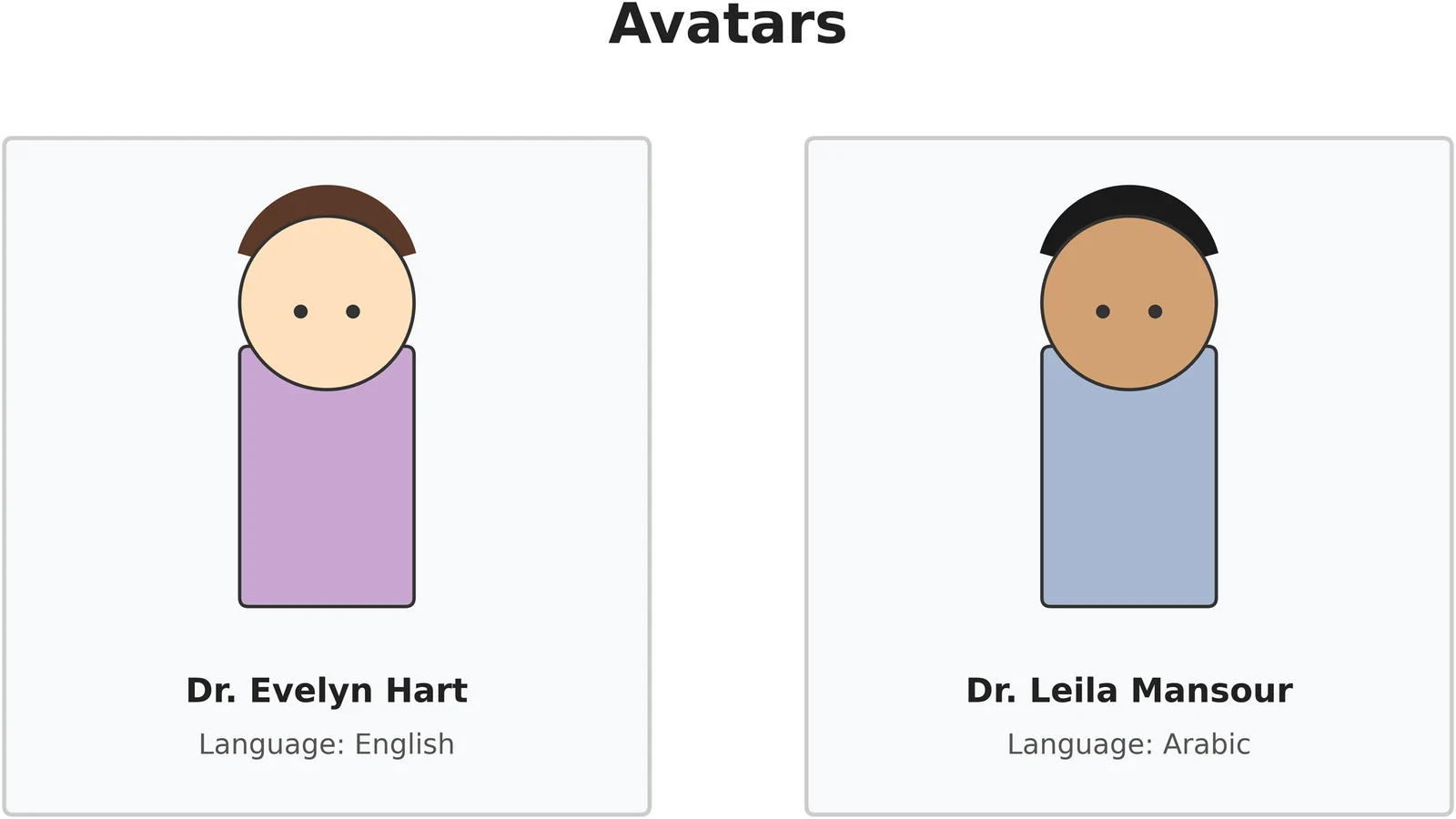
Avatar Design Schematic icons representing the two virtual therapist avatars — one for English-speaking users, one for Arabic-speaking users — used in the VR intervention system, supporting language and cultural accessibility. Original diagram; not MetaHuman Creator renders.

This thoughtful character design ensures the avatar not only acts as a responsive interface but also reinforces the immersive and therapeutic goals of the VR system. Schematic representations of the English-speaking and Arabic-speaking avatars are shown in Fig 7.

## Results and discussion

### Machine learning performance

The performance of both models, XGBoost and LightGBM, were evaluated to assess their effectiveness in classifying pain levels from EEG signals. Both models were trained on an identical dataset, which was composed of various features derived from the EEG channels. Since the pain levels were discrete values, the classification task was treated as a multi-class problem. Table 5 summarizes the classification results obtained from both models. Metrics including accuracy, precision, recall and F1-score (all macro-averaged) were utilized for a comprehensive assessment of the model performance. Cross validation techniques were applied for each model before testing it on the 20% testing subset of the data. The performance metrics of both models are illustrated in Table 5.

**Table 5:** Performance Comparison Between XGBoost and LightGBM Models.

Model	Accuracy	Cross Validation Accuracy	Precision	Recall	F1-Score
LightGBM	0.9789	0.9578 (±0.82%)	0.99	0.99	0.99
XGBoost	0.9725	0.9609 (±0.70%)	0.98	0.97	0.97

The results above indicate that both of the models performed comparably well, attaining high accuracy and robust generalization across the cross-validation folds. LightGBM demonstrated slightly higher values in test accuracy, precision, recall, and F1-score in comparison to XGBoost, but the differences were not statistically significant. On the other hand, XGBoost had a more consistent cross-validation performance. The marginal differences between the two models suggest that both frameworks are highly effective for EEG-based pain classification tasks.

Table 6 compares the results of this study with existing state of the art methods in pain classification, highlighting competitive performance. The results of our study demonstrate the robustness of our models, for the models exhibited strong accuracy across various pain levels (0–10). This contrasts sharply with other studies, where the pain classes are either binary classes (no pain, pain) [9,28] or up to 5 classes [8,10,29–31]. Thus, this comparative analysis illustrates our study’s finer granularity in pain classification and allows for better clinical integration, especially in contexts where multi-class classification is preferred.

**Table 6:** Comparison of classification performance with existing literature.

Study	Model	Accuracy	Classes
Al-Nafjan et al. [8]	CNN, RNN	87.94%	3 (low, moderate, high)
Mari et al. [9]	Random Forest	73.18%	2 (low, high)
Elsayed et al. [10]	ANN	94.83%	4 (no pain, low, moderate, and high)
Afrasiabi et al. [29]	Bayes-optimized SVM	93.33%	5 (no pain, low, moderate, high, intolerable)
Nezam et al. [30]	SVM, KNN (with decision tree)	SVM: 83% (3-class), 62% (5-class) KNN: 80% (3-class), 60% (5-class)	3, 5 (no pain, 1st-4th level)
Bonotis et al. [31]	Stochastic Forest	72.7%	5 (no pain, low, mid, high, unbearable)
Wang et al. [28]	Auto-encoder and Logistic Regression	74%	2 (high and low)
**This Study**	XGBoost / LightGBM	LightGBM: 97.89% XGBoost: 97.25%	11 (pain levels 0–10)

In addition, the existing literature explored various approaches, including machine learning and deep learning based techniques. The ML approaches [9,29–31] have achieved accuracies as low as 60% and as high as 93.33%, whereas DL techniques [8,10] have obtained accuracies ranging from 87.94% to 94.83%. A hybrid approach [28] is also observed, where an accuracy of 74% was reported. Based on this, it can be inferred that our algorithms have shown a substantial improvement in comparison to the other studies.

It is noted that our LightGBM and XGBoost models have exhibited accuracies of 97.89% and 97.25%. Comparing their performance with the existing studies, our models outperform the best classical ML result of 93.33% [29] by 4.56% and 3.92%, respectively. Similarly, compared to the highest-performing DL model [10], LightGBM demonstrates an improvement of 3.06%, whereas XGBoost by 2.42%. Furthermore, taking into consideration the average accuracy across ML-based studies (approximately 74.88%), both of our models show a refinement of over 20 percentage points. This high performance, while classifying 11 distinct pain levels (0–10), represents a notable jump in performance even when dealing with a much higher number of classes than seen in usual literature. It is also a testament to the robustness of our machine learning pipeline, which significantly minimized noise and maximized feature discriminability. Specifically, the combination of rigorous data preprocessing (e.g., ICA and AutoReject) and the use of wavelet-based features provided the models a highly refined and representative feature set, allowing them to distinguish precise decision boundaries for multi-class classification, despite the increased complexity.

These insights convey practicality. Our models entail less computational resources and faster training time, in comparison to deep learning models that require large datasets and intensive tuning. This makes our models more feasible for real-time clinical applications. Overall, both models are proven to be effective in multi-class pain classification based on EEG data. Considering the similar performance demonstrated by both models, the decision between XGBoost and LightGBM would depend on the factors impacting real-world applications, including computational efficiency, scalability, and integration.

### Functional demonstration and pain level visualization

The functional demo validated the full closed-loop pipeline of our AI-powered VR therapy system, demonstrating real-time interaction between EEG-based pain prediction and responsive avatar behavior in Unreal Engine. Through a local server and the UWS Plugin, EEG data processed by XGBoost or LightGBM was converted into categorized pain levels, triggering distinct behavioral states in the virtual environment.

Using Unreal Engine 5.3.2, we developed a controlled VR setting centered around our MetaHuman avatar, Dr. Evelyn Hart, who adapted her verbal and non-verbal responses depending on the severity of detected pain. Blueprint scripting was used to build logic gates that map incoming pain levels to specific animations, facial expressions, and voice lines, ensuring timely therapeutic feedback.

The pain categories are as follows.

1 Slight Pain (1–3)

(a) Avatar Response: Calm encouragement with gentle expressions and soft speech. The avatar delivers a calm message and uses mindfulness techniques to comfort the user in a peaceful forest environment.
(b) Environment Description: A tranquil forest environment with birdsong and trees gently swaying. No urgent visual cues, supporting a peaceful mood.
(c) Timing Setup: EEG-derived pain level is sent every 60 seconds during test simulation.

**Fig 8:**
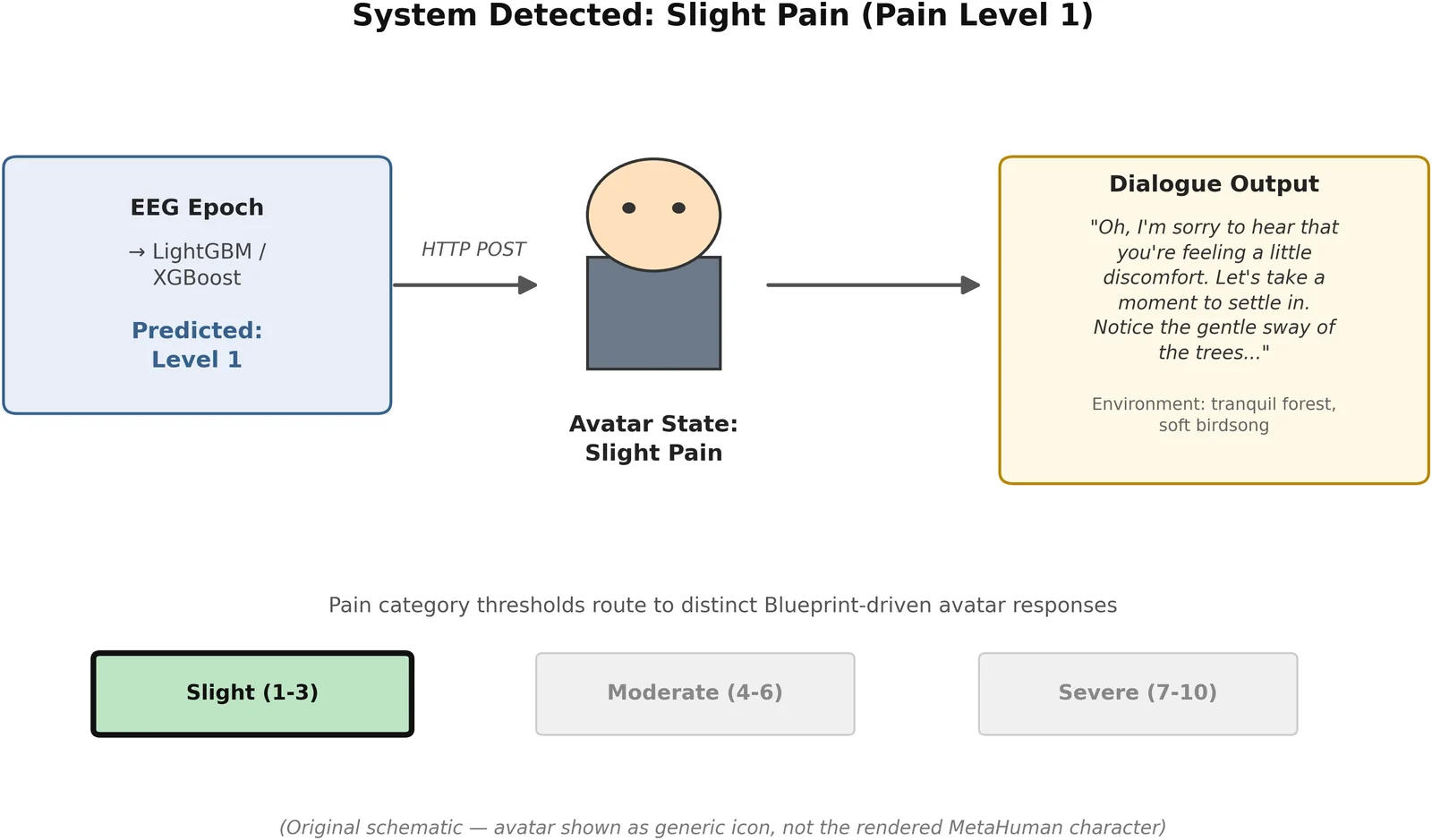
System Detected Slight Pain (Pain Level 1) The avatar displays a mild response corresponding to a pain level 1 prediction by the trained machine learning model. This indicates the system’s sensitivity in recognizing low-intensity pain based on EEG input. Original diagram, not a rendered engine screenshot.

Fig 8 highlights the system response to slight pain.

2 Moderate Pain (4–6)

(a) Avatar Response: The avatar acknowledges increased discomfort, maintaining a caring tone. She guides the patient through deeper breathing and visualization.
(b) Environment Description: Scene shifts slightly with soft wind audio, ambient lighting, and more direct avatar eye contact.
(c) Timing Setup: Moderate-level updates are sent every 60 seconds from the prediction script.

**Fig 9:**
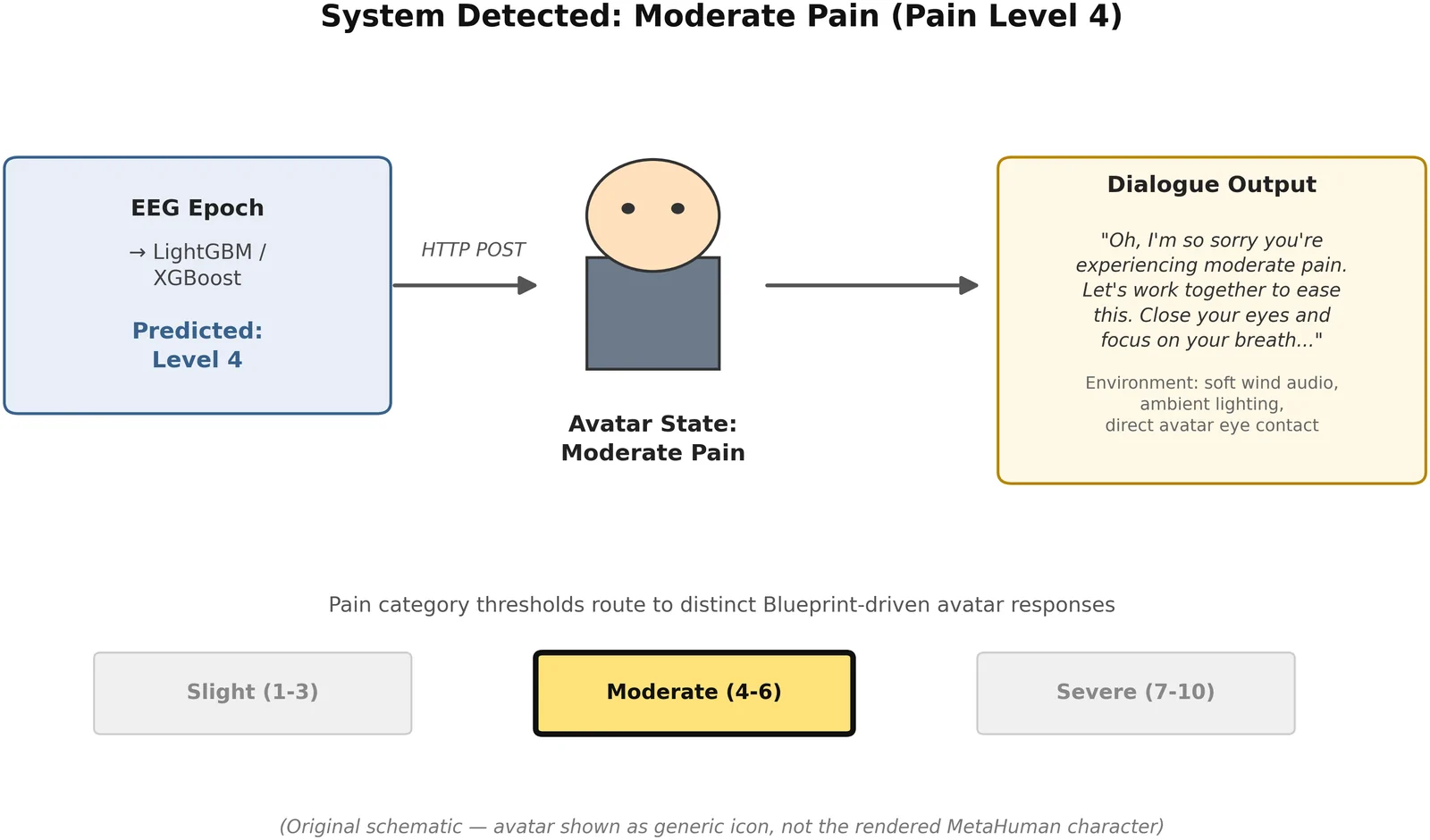
System Detected Moderate Pain (Pain Level 4) The avatar exhibits a noticeable reaction corresponding to a predicted pain level 4, demonstrating the system’s capability to detect and reflect mid-range pain intensity in real time. Original diagram, not a rendered engine screenshot.

Fig 9 highlights the system response to moderate pain.

3 Severe Pain (7–10)

(a) Avatar Response: Avatar shows strong emotional empathy. Eyes widen, tone becomes urgent yet reassuring.
(b) Environment Description: Avatar leans forward with animated gestures. Slight visual dimming in the environment to reflect urgency.
(c) Timing Setup: EEG data is transmitted once every minute, just like other levels.

**Fig 10:**
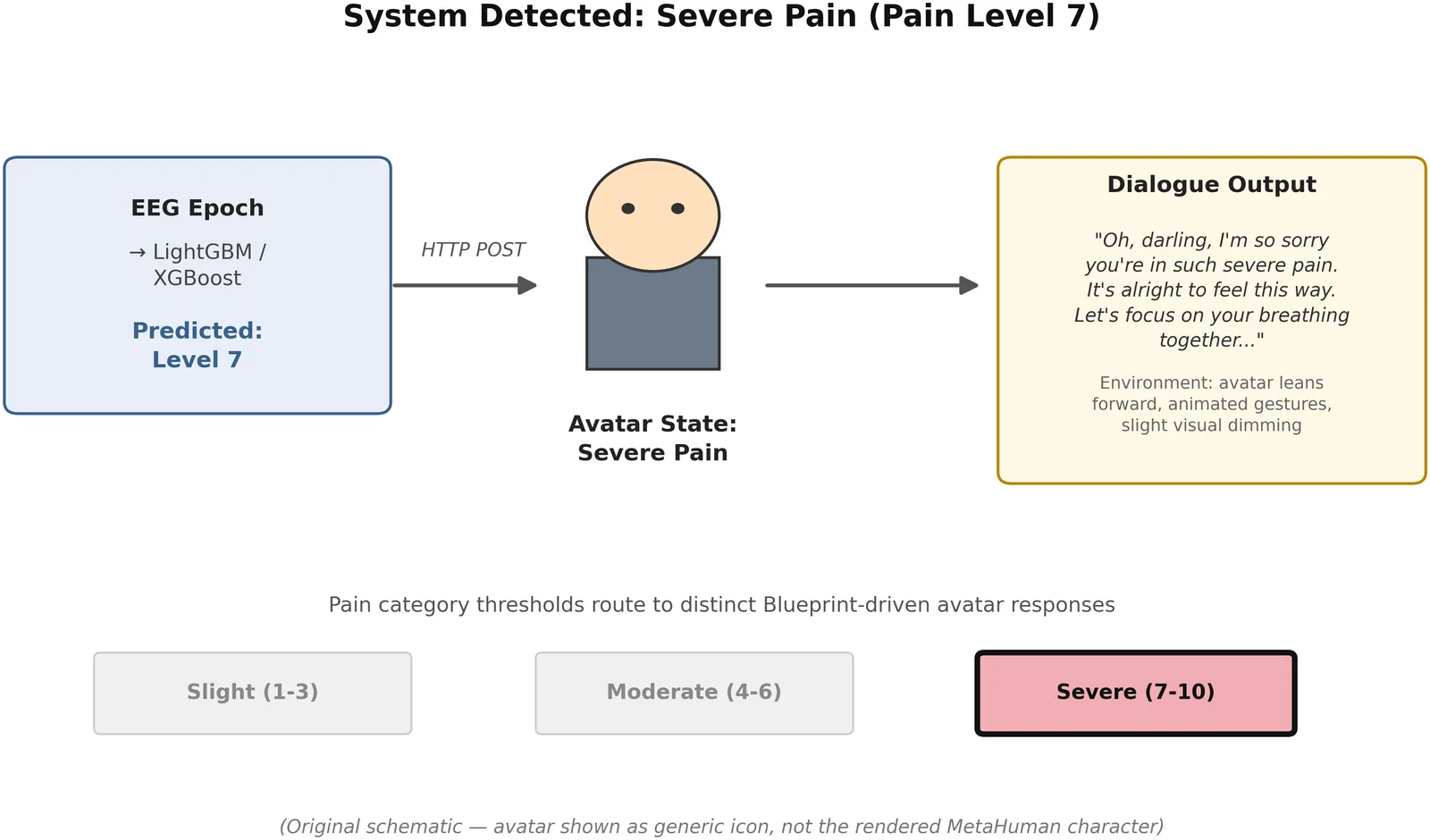
System Detected Severe Pain (Pain Level 7) The avatar displays a strong visual response to a predicted pain level 7, representing high pain intensity and validating the system’s sensitivity to critical pain states. Original diagram, not a rendered engine screenshot.

Fig 10 highlights the system response to severe pain.

The transition between pain levels was seamless in real-time, as predictions were streamed through HTTP POST requests and interpreted instantly by Unreal’s Blueprint logic. This confirms the reliability of both the communication protocol and the responsiveness of the avatar system.

The integration of categorized pain reactions within Unreal Engine not only makes the therapy immersive and personalized but also provides a template for future enhancements, such as emotional co-tracking, haptic integration, or multi-language support.

### VR blueprint integration and system workflow

To enable real-time interaction between the machine learning pain predictions and the MetaHuman avatar’s behavior, we implemented a custom Blueprint logic system within Unreal Engine 5.3.2. Blueprints are Unreal Engine’s visual scripting language, allowing rapid development of game logic without requiring C++ coding.

Our blueprint system operates as the reactive engine of the therapeutic environment. It listens for incoming JSON data from the Python server via the UWS plugin, parses the predicted pain level, and uses conditional nodes to drive the appropriate avatar response. The core blueprint logic flow can is described below.

1. HTTP Data Reception: The system receives real-time HTTP POST requests containing a pain level value ranging from 0 to 10. This is handled through a custom event bound to the UWS plugin interface.
2. Pain Level Parsing & Categorization: The blueprint parses the incoming value and routes it through a switch-case-like structure, mapping it into one of three categories: Slight (1–3), Moderate (4–6), or Severe (7–10).
3. Avatar Reaction Triggering Based on the category:
4. A facial animation sequence is triggered (e.g., calm smile for slight, concerned frown for severe).
5. A voice line is selected from pre-recorded audio assets linked to the pain level.
6. Gestural animations such as nodding, hand raises, or leaning in are also activated to enhance emotional realism.
7. Fallback and Idle Handling: If no data is received for a specified time (e.g., 10 seconds), the blueprint reverts the avatar to an idle loop using subtle animations like blinking or soft breathing to maintain realism without overwhelming the user.

**Fig 11:**
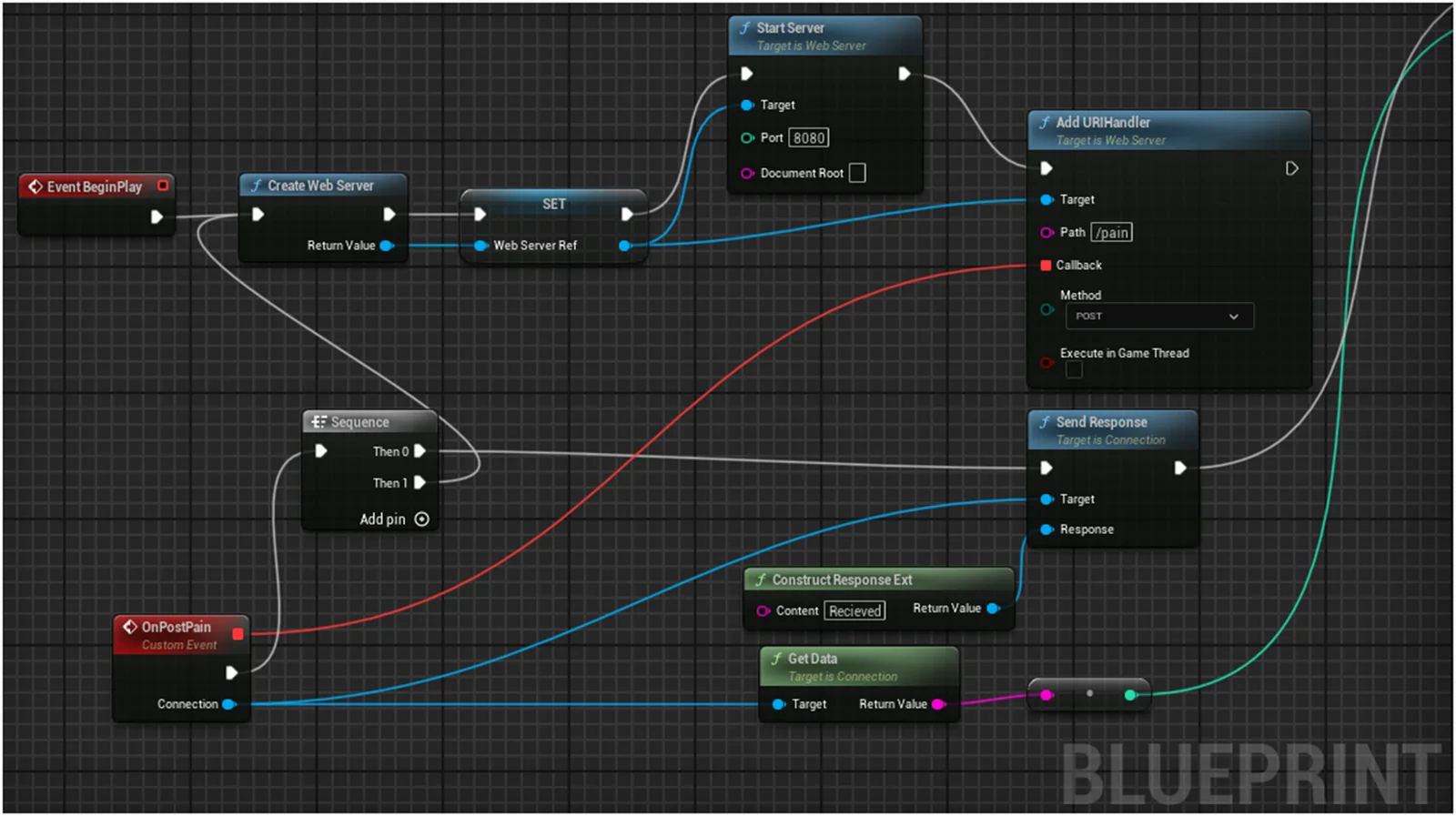
Blueprint Logic For Different Pain Categories Blueprint logic handling pain category differentiation. The system routes incoming pain level values to corresponding avatar responses based on categorized thresholds: slight (1–3), moderate (4–6), and severe (7–10).

**Fig 12:**
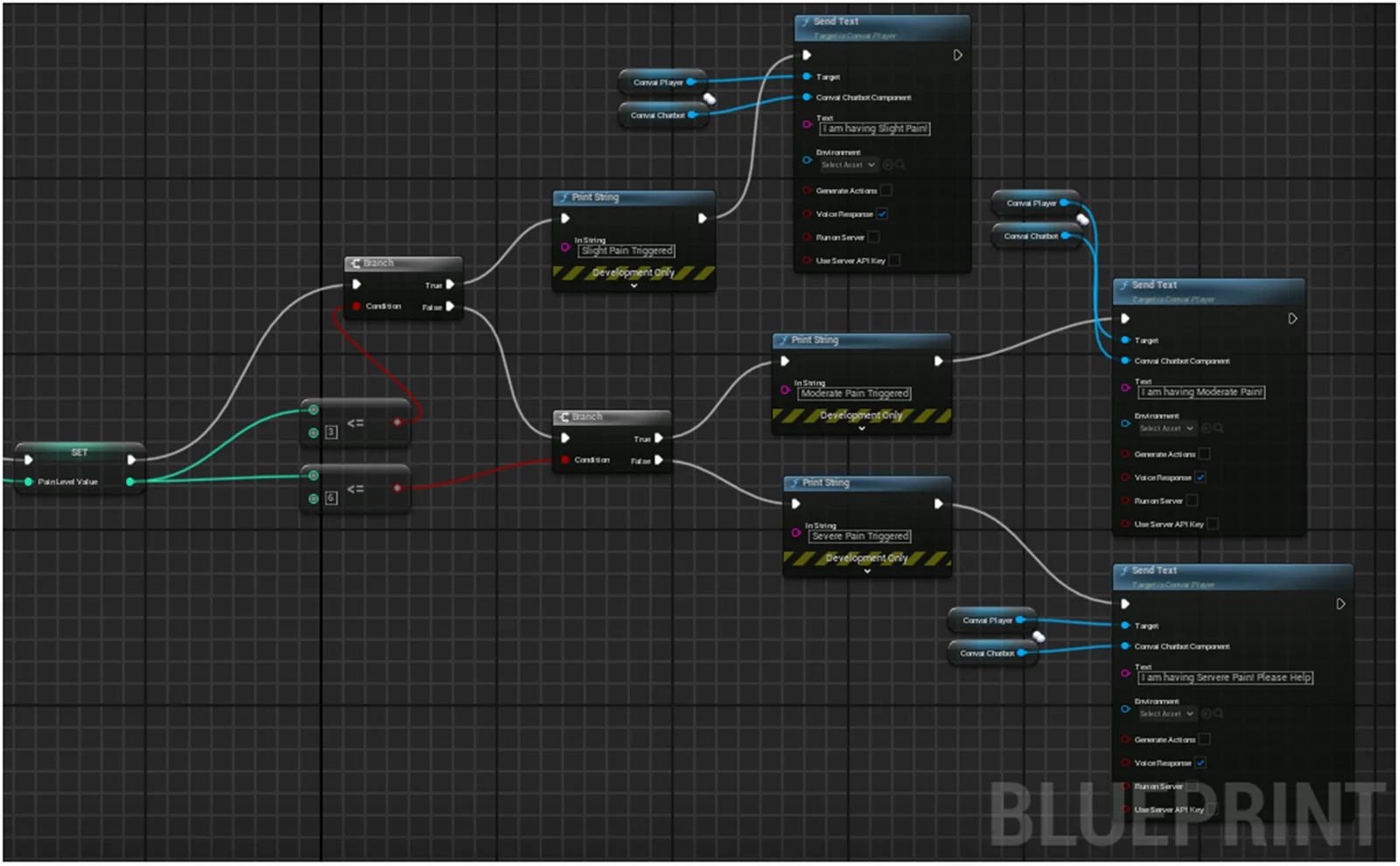
Blueprint For Server Logic (Starting up Server When Unreal Engine Runs) Blueprint logic for initializing the server on Unreal Engine startup. This setup enables automatic activation of the HTTP listener to receive real-time pain level data.

As shown in Fig 11 and 12, this modular Blueprint structure ensures flexibility in updating or adding new pain categories, emotional states, or behavioral logic without rewriting backend code. It also makes the system scalable, maintainable, and ideal for future clinical scenarios requiring adaptive, context-aware avatar responses.

### Strengths

The study highlights several strengths. First, the closed-loop integration of EEG-based pain classification with a real-time VR therapeutic environment is a novel and technically demanding contribution that bridges neuroscience, artificial intelligence, and immersive technology. Second, the machine learning pipeline achieves high accuracies (97.89% LightGBM; 97.25% XGBoost) across 11 discrete pain levels, enabling clinically meaningful fine-grained monitoring. Third, unlike computationally intensive deep learning alternatives, the XGBoost and LightGBM models are lightweight, interpretable, and CPU-deployable, making them practical for real-world applications with limited resources. Fourth, the system supports both English and Arabic-speaking users through dual-language avatar configurations, enhancing cultural accessibility. Fifth, the modular blueprint-driven architecture allows seamless integration of future extensions such as emotional state tracking, haptic feedback, or reinforcement learning-based personalization without requiring rewriting of core system components.

### Limitations

There are several limitations in this current study that should be acknowledged. First, the machine learning models were trained and evaluated exclusively on a pre-recorded, open-source EEG dataset, meaning live EEG signal acquisition from real patients in a VR session has not yet been validated. Second, the current cross-validation strategy involves random shuffling of all samples prior to fold assignment, which may introduce within-subject data leakage; training and testing folds may contain epochs from the same participants. This may inflate reported accuracy and limit the generalizability of the models to unseen patients. Third, hardware constraints, including limited RAM on the initial development machine, caused rendering instability in the VR environment. This necessitated asset simplification and lighting adjustments. Lastly, the system has not yet undergone clinical trials with chronic pain patients, limiting the assessment of its therapeutic efficacy in real-world settings.

### Implications

The findings of this study carry significant implications for the field of non-pharmacological pain management. By demonstrating that EEG signals can be interpreted in real time to drive adaptive, personalized VR-based therapeutic interventions, this work establishes a credible and technically feasible framework for drug-free analgesia. Clinically, the system has direct applicability in settings where patients experience chronic or procedural pain, including oncology, rehabilitation, post-operative care, and burn treatment. The dual-language capability (English and Arabic) underscores the system’s potential for deployment in diverse cultural and linguistic contexts. From a research perspective, the study highlights that gradient boosting models, despite being less fashionable than deep learning approaches, remain highly competitive for EEG-based biosignal classification tasks, particularly under the data scarcity and computational constraints typical of clinical EEG environments. The SHAP-based interpretability framework also opens pathways for clinical trust and regulatory consideration by making AI predictions transparent to clinicians.

### Recommendations

Based on the findings and limitations of this study, the following recommendations are proposed for future research:

1. Subject-Wise Cross-Validation: Before clinical deployment, the cross-validation strategy should be transitioned to a subject-wise k-fold split, ensuring that training and testing folds are composed of data from entirely different participants. This is critical for an accurate assessment of model generalizability to unseen patients.
2. Live EEG Integration: Future iterations should incorporate real-time EEG signal acquisition from wearable headsets to validate the full closed-loop pipeline with live data.
3. Clinical Trials: The system should undergo controlled clinical trials with patients experiencing pain to empirically assess its therapeutic efficacy, patient adherence, and safety profile. Patient feedback should be used to iteratively refine avatar dialogue, environmental design, and pain threshold calibration.
4. Emotionally Adaptive Responses: The integration of real-time emotion recognition through facial expression tracking and voice analysis would enable the avatar to respond to the patient’s emotional state in addition to their pain level, creating a more holistic therapeutic interaction.
5. Multi-Avatar Therapy System: Expanding the system to offer a selection of virtual therapist avatars with distinct personalities, appearances, and interaction styles could improve patient engagement and therapeutic adherence across diverse demographic groups.
6. Extended Applications: The modular architecture of this system lends itself to extension beyond pain management into broader mental health therapy domains, such as anxiety, PTSD, and emotional dysregulation.

## Conclusion

The study successfully designed, developed, and integrated an end-to-end AI-powered virtual reality therapy system from scratch. It combines real-time EEG data preprocessing, machine learning-based pain prediction, and immersive avatar-guided therapy into a unified, closed-loop framework. Using XGBoost and LightGBM models, the system accurately classifies pain levels from EEG features and relays these predictions to a VR environment developed in Unreal Engine 5.3.2. Within this environment, a MetaHuman avatar responds to the predicted pain levels through facial expressions, dialogue, and environmental cues, offering patients a personalized, drug-free therapeutic experience. The complete pipeline was built using Python for data processing and modeling, and Unreal Engine blueprints with ConvAI integration for dynamic avatar behavior and speech.

This marks the successful completion of the first operational phase. All critical modules, from EEG signal collection and pain classification to real-time VR feedback, were independently developed, validated, and integrated into a functioning prototype. The outcome demonstrates the technical feasibility and clinical potential of closed-loop, EEG-driven VR therapy. With this solid foundation in place, future phases will focus on expanding emotional recognition, optimizing system performance, conducting clinical trials, and enhancing personalization through reinforcement learning. The system holds strong promise as a next-generation therapeutic platform that bridges neuroscience, artificial intelligence, and immersive technology for non-invasive pain management.

### Contributions of the study

This study presents a prototype of a fully integrated, real-time, EEG-driven virtual reality (VR) pain therapy system. Unlike prior work relying on passive VR exposure or offline EEG analysis, our system closes the loop between biosignal data input, machine learning-based pain classification, and immersive therapeutic feedback within a unified pipeline. The key novel contributions of this work include:

1. High-Resolution Pain Classification: Fine-grained classification of 11 discrete pain levels (0–10) from EEG features—the highest class resolution reported in comparable literature—utilizing computationally efficient gradient boosting models (XGBoost and LightGBM) optimized for real-time clinical deployment.
2. Emotionally Responsive Virtual Therapist: An interactive MetaHuman avatar that dynamically adapts its dialogue, facial expressions, and body language in response to the user’s predicted pain severity.
3. Cross-Cultural Accessibility: Dual-language support for both English- and Arabic-speaking users, significantly broadening the system’s clinical and cultural reach.

Together, these advancements represent a major step toward practical, non-pharmacological, and personalized pain management powered by neuroscience and artificial intelligence.
